# Neurofascin Is a Novel Component of Rod Photoreceptor Synapses in the Outer Retina

**DOI:** 10.3389/fncir.2021.635849

**Published:** 2021-02-10

**Authors:** Sahar Pourhoseini, Debalina Goswami-Sewell, Elizabeth Zuniga-Sanchez

**Affiliations:** ^1^Department of Ophthalmology, Baylor College of Medicine, Houston, TX, United States; ^2^Department of Neuroscience, Baylor College of Medicine, Houston, TX, United States

**Keywords:** retina, synapse, rods, rod bipolars, neurofascin, cell adhesion molecule

## Abstract

Neural circuit formation is an intricate and complex process where multiple neuron types must come together to form synaptic connections at a precise location and time. How this process is orchestrated during development remains poorly understood. Cell adhesion molecules are known to play a pivotal role in assembling neural circuits. They serve as recognition molecules between corresponding synaptic partners. In this study, we identified a new player in assembling neural circuits in the outer retina, the L1-family cell adhesion molecule Neurofascin (Nfasc). Our data reveals Nfasc is expressed in the synaptic layer where photoreceptors make synaptic connections to their respective partners. A closer examination of Nfasc expression shows high levels of expression in rod bipolars but not in cone bipolars. Disruption of Nfasc using a conditional knockout allele results in selective loss of pre- and post-synaptic proteins in the rod synaptic layer but not in the cone synaptic layer. Electron microscopic analysis confirms that indeed there are abnormal synaptic structures with less dendrites of rod bipolars innervating rod terminals in loss of Nfasc animals. Consistent with these findings, we also observe a decrease in rod-driven retinal responses with disruption of Nfasc function but not in cone-driven responses. Taken together, our data suggest a new role of Nfasc in rod synapses within the mouse outer retina.

## Introduction

**Figure 1:**
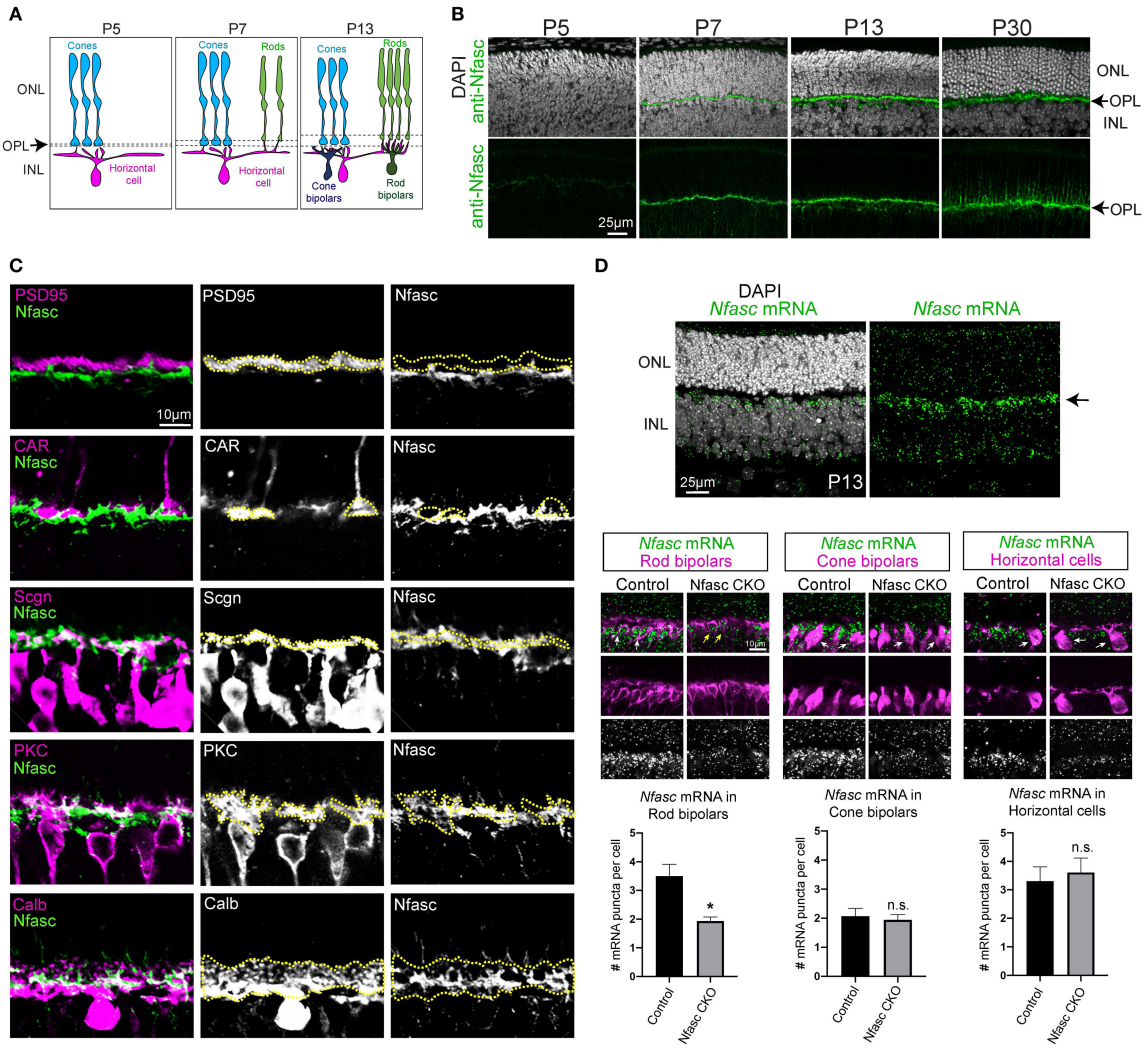
Nfasc is expressed in the OPL during synapse formation **(A–D)**. Schematic drawing of outer retinal development. Cones (blue) initially make contact to horizontal cells (magenta). At P7, rods begin to contact horizontal cells. By P13, cone bipolars (dark blue) and rod bipolars (dark green) located in the inner nuclear layer (INL) form synapses with cones and rods, respectively **(A)**. Antibody staining in wild-type retinas reveals Nfasc (green) is not detected at P5 and only becomes visible until P7 when the OPL emerges. Nuclei is stained with DAPI **(B)**. Co-labeling of Nfasc with known cell-type specific markers **(C)**. Rod terminals are stained with anti-PSD-95 (magenta) and show little overlap with anti-Nfasc (green) as depicted with yellow dotted traces. Cone terminals (anti-CAR, magenta) also shows little co-localization with Nfasc (green). Nfasc appears to be expressed in the same layer as the dendrites of cone bipolars (anti-Scgn) and rod bipolars (anti-PKC) as well as with the processes of horizontal cells (anti-Calb) **(C)**. *Nfasc* mRNA (green) is detected by *in situ* hybridization in wild-type retinas at P13 and shown to localize to the top-most region of the INL. Antibody staining after *in situ* hybridization shows *Nfasc* mRNA expression in rod bipolars (anti-PKC), cone bipolars (anti-Scgn), and horizontal cells (anti-Calb). Quantification of *Nfasc* mRNA puncta (0.6 μm in size) per cell shows Nfasc is significantly reduced in rod bipolars but not in cone bipolars nor horizontal cells in Nfasc CKO. Data are represented as mean values ± SEM. Statistical significance determined by an unpaired two-tailed Student's *t*-test. *p < 0.05. Images are shown as confocal sections. Scale bar shown in each figure panel.

Throughout the developing nervous system, neurons form synaptic connections to distinct targets. However, the key molecules that instruct neurons to select their appropriate partners remains poorly understood. The mouse outer retina is an excellent system to study how neurons chose their appropriate synaptic targets (Sanes and Zipursky, 2010, 2020). First, all the neuron types and their respective connections have been well-characterized (Dunn and Wong, 2012; Behrens et al., 2016; Shekhar et al., 2016). Second, the retina is a highly organized laminated structure where connectivity defects often result in disorganization of the synaptic layer (Dick et al., 2003; Soto et al., 2013; Ribic et al., 2014). And third, neurons in the outer retina extend their dendrites and axon in a relative short distance, as their synaptic partners are located in close proximity (Sarin et al., 2018). In the outer retina, rod and cone photoreceptors reside in the Outer Nuclear Layer (ONL) and they send their axon into the Outer Plexiform Layer (OPL) where they synapse to distinct interneurons (Hoon et al., 2014; Zhang et al., 2017; Sarin et al., 2018). These interneurons include horizontal cells and bipolar cells, which are located in the Inner Nuclear Layer (INL). See Figure 1A. Horizontal cells connect laterally to the different photoreceptors where the dendrites connect to cones and the axon to rods (Kolb, 1974). Cones contact horizontal cells around postnatal day (P) 3-4, whereas rods synapse to the axon of horizontal cells at P7-8 (Olney, 1968; Blanks et al., 1974; Rich et al., 1997; Sarin et al., 2018). Following horizontal cell connectivity, photoreceptors then synapse to their respective bipolar cell (i.e., rod bipolars and cone bipolars) as shown in Figure 1A. Dendrites of both rod bipolars and cone bipolars extend into the OPL where they innervate rods and cones, respectively (Haverkamp et al., 2000; Dunn and Wong, 2012; Euler et al., 2014; Behrens et al., 2016). Cone bipolars make synapses with cones around P7, and rod bipolars synapse with rods at P9 (Sherry et al., 2003; Sarin et al., 2018; Anastassov et al., 2019). Synapse formation is largely complete by P21.

Cell adhesion molecules mediate key molecular interactions among different neuron types (Zipursky and Sanes, 2010; Sanes and Zipursky, 2020). They serve as molecular cues in pre- and post-synaptic neurons to establish proper connectivity among appropriate synaptic partners (Zipursky and Sanes, 2010; Sanes and Zipursky, 2020). Photoreceptor wiring is a multi-step process. As described above, initial contacts are made between photoreceptors and horizontal cells followed by contacts between photoreceptors and bipolar cells (Olney, 1968; Blanks et al., 1974; Rich et al., 1997; Sarin et al., 2018). Several cell adhesion molecules have been implicated at different stages during photoreceptor connectivity. The cell adhesion molecules SynCAM1 and Netrin-G ligand-2 (Ngl-2) are important in rod-to-horizontal cell connectivity where loss of SynCAM1 or Ngl-2 results in processes from horizontal cells failing to be confined to the synaptic layer, and instead misproject into the ONL (Soto et al., 2013; Ribic et al., 2014). Similarly, members of the Sema and Plexin family are also implicated in rod-to-horizontal cell connectivity where loss of Sema6a or its receptor PlexinA4 disrupt the normal positioning of horizontal cell processes within the OPL (Matsuoka et al., 2012). In rod-to-rod bipolar connectivity, the leucine-rich repeat cell adhesion molecule Elfn1 is found in rods and is critical in recruiting pre- and post-synaptic machinery to the OPL where Elfn1 binds trans-synaptically to the metabotropic Glutamate Receptor 6 (mGluR6) expressed in rod bipolars (Cao et al., 2015; Wang et al., 2017). Another cell adhesion molecule implicated in rod-to-rod bipolar connectivity is Dystroglycan, which is expressed in rods and binds to the extracellular matrix protein, Pikachurin (Omori et al., 2012). Loss of Dystroglycan or Pikachurin results in rod bipolars failing to invaginate rod terminals and recruit components for phototransduction (Omori et al., 2012; Orlandi et al., 2018). These findings highlight how cell adhesion molecules are critical for synapse formation; however, only a few of these molecules have been uncovered in the outer retina.

To identify novel cell adhesion molecules involved in synaptogenesis, we looked for unique expression patterns of candidate genes using published RNA sequencing data of mouse outer retinal neurons during synaptogenesis (Sarin et al., 2018). From our analysis, we find the L1-family cell adhesion molecule Neurofascin (Nfasc) to be a promising candidate as Nfasc is expressed throughout synapse formation (Sarin et al., 2018). In the present study, we found Nfasc to be highly expressed in rod bipolars. Moreover, loss of Nfasc results in synaptic defects in rod to rod bipolar connectivity. This is seen by (i) a reduction of synaptic protein expression only in the rod synaptic layer, (ii) less dendrites of rod bipolars contacting rod terminals, and (iii) a decrease of rod-driven retinal responses. Based on these data, we propose Nfasc is a novel molecule important for synapses between rods and rod bipolars.

## Materials and Methods

### Animals

All mouse procedures were approved by Baylor College of Medicine Institutional Animal Care and Use Committee. *Nfasc*^*flox*/*flox*^ mice removes exon 4 as described in Amor et al. (2017) and were kindly provided by Dr. Matthew Rasband. *Chx10cre* mice have been described in Rowan and Cepko (2004) and were generously provided by Dr. Melanie Samuel. To conditionally remove Nfasc function in the retina, we crossed *Chx10cre; Nfasc*^*flox*/*wt*^ transgenic mice with *Nfasc*^*flox*/*flox*^ which we refer to as Nfasc CKO. *Nfasc*^*flox*/*flox*^ mice without *Chx10cre* served as controls. Wild-type retinas are from CD1 mice purchased from Charles River. Both males and females were used in experiments.

### Immunohistochemistry

Eyes were collected at various developmental time points with P0 designated as the day of birth. Whole eyes were fixed at 60 min in 4% paraformaldehyde in PBS except for mGluR6 staining which were lightly fixed at room temperature for 10 min. Eye cups were dissected and sectioned at 20 μm as previously described (Sarin et al., 2018). Slides were dried overnight and washed with PBS for 10 min twice to start antibody staining. Sectioned slides were incubated with blocking buffer (10% normal goat serum, 1% BSA, 0.5% Triton X-100 in PBS) followed by primary antibodies at 4°C overnight (see Table 1). Slides were washed 3 times with PBS for 10 min each and then incubated with secondary antibodies at 1:1000 dilution at 4°C overnight. Slides were then washed 3 times with PBS, stained with DAPI (1:1000), and then sealed with Vectashield (Vector Laboratories).

**Table 1:** List of primary antibodies used in this study.

**Antibody name**	**Labeling specificity**	**Source**	**Dilution**
Chicken polyclonal anti-Nfasc	Neurofascin	R&D Systems Cat#AF3235, RRID:AB_10890736	1:1000
Rabbit anti-Cone Arrestin (CAR)	Cone Photoreceptors	Millipore Cat# AB15282, RRID:AB_1163387	1:500
Mouse monoclonal anti-Bassoon	Presynaptic photoreceptor terminals	Enzo Life Sciences Cat# VAM-PS003F, RRID:AB_1659573	1:500
Rabbit anti-Calbindin (Calb)	Horizontal cells, amacrine cells, retinal ganglion cells	Swant Cat# CB38, RRID:AB_10000340	1:2000
Mouse monoclonal anti-Protein Kinase C (PKC)	Rod bipolar cells	Abcam Cat# ab31, RRID:AB_303507	1:500
Mouse polyclonal anti-PSD-95	Photoreceptor terminals (highly expressed in rods compared to cones)	Thermo Fisher Scientific Cat# MA1-046, RRID:AB_2092361	1:500
Rabbit polyclonal anti-metabotropic Glutamate Receptor 6 (mGluR6)	ON biopolar cells	Gift from Larry Zipursky; (Sarin et al., 2018)
Rabbit polyclonal anti-Secretagogin (Scgn)	Cone bipolar cells	BioVendor Laboratory Medicine Cat# RD181120100, RRID:AB_2034060	1:1000
Mouse monoclonal anti-CtBP2	Photoreceptor terminals	BD Biosciences Cat# 612044, RRID:AB_399431	1:500

### RNAscope

We performed *in situ* hybridization at P13 using RNAscope technology (Advanced Cell Diagnostics) and following manufacturer's instructions. To detect *Nfasc* mRNA, we used Mm-Nfasc-C2 (Cat#558151-C2) probe on 20 μm retinal sections followed by antibody staining as described above to visualize horizontal cells (anti-Calb), rod bipolars (anti-PKC), and cone bipolars (anti-Scgn). Quantification of *Nfasc* mRNA puncta was performed using the confocal imaging software Imaris version 9.6 (Bitplane, South Windsor, CT, USA). The number of Nfasc puncta (0.6 μm in size) was detected using the Imaris Spot feature. Quantification of *Nfasc* puncta within the cell bodies of rod bipolars, cone bipolars, and horizontal cells was done in at least two retinal sections from one control mice and from two Nfasc CKO animals.

### Histological Quantification

For quantification, images were collected from 3 to 4 animals per group with at least 3 confocal images per animal taken with the same confocal settings. Nfasc antibody staining was performed in all retinal sections to confirm loss of Nfasc in Nfasc CKO. All images were taken from roughly the same position in the retina (central-periphery) where we noticed significant reduction in Nfasc protein expression. Confocal images were acquired using a Zeiss LSM 800 microscope and then analyzed with Imaris. Quantification of pre- and post-synaptic marker expression was performed using the Imaris Spot feature. Puncta with a diameter of 0.6 μm for Bassoon, 0.7 μm for CtBP2, and 1 μm for mGluR6 were counted in retinal sections. These measurements were performed in *n* = 30–40 retinal sections (from 10 different confocal sections) from four different control animals and three different Nfasc CKO mice. The number of Bsn within cone terminals were averaged from 3 retinal slices from controls (*n* = 4 mice) and Nfasc CKO (*n* = 4 mice). Retinal layer thickness was measured in confocal sections stained with DAPI using Imaris. Statistical significance between experimental groups and controls was determined using an unpaired two-tailed Student's *t*-test. All statistical analysis was performed using GraphPad Prism version 9 with *p*-values given in the text and figure legends.

### Transmission Electron Microscopy (TEM)

Eye cups were fixed in 3% glutaraldehyde in 4°C overnight. Tissue samples were washed in 1M sodium phosphate buffer (pH 7.3), post-fixed in 1% osmium tetroxide for 1 h and dehydrated through a series of graded alcohol steps. Tissue samples were infiltrated (harden) with acetone and polybed 812 plastic resin and embedded in plastic block molds with 100% polybed 812. Ultra-thin sections (80 nm) were cut using a Leica EMUC ultra microtome at the same central-periphery location where we detect minimal Nfasc expression based on immunohistochemistry. These sections were mounted on 100 mesh copper grids and stained with 2% uranyl acetate and Reynold's lead stain. Grids were visualized on a JEOL JEM 1230 electron microscope and images were captured on an AMTV600 digital camera. Analysis was performed on 3–4 different TEM images taken from four controls and four Nfasc CKO mice. ImageJ software was used to measure ribbon length within triad structures. Statistical significance was determined using an unpaired Student's *t*-test.

### Electroretinography (ERG)

Scotopic ERGs were recorded bilaterally from four control animals (*n* = 8) and four Nfasc CKO mice (*n* = 8) at 4–5weeks old. Mice were dark adapted overnight and anesthetized with a weight-based i.p. injection solution of ketamine (46 mg/ml), xylazine (9.2 mg/ml), and acepromazine (0.77 mg/ml). Pupils were dilated with a drop of 1% tropicamide and 2.5% phenylephrine. Mice were placed on a heating pad and a single drop of 2.5% methylcellulose gel was applied on each eye before placing a platinum electrode in contact with the center of the eye. Similar platinum electrodes were placed at the base of the tail and another between the ears to serve as ground and reference electrodes, respectively. Mice were moved into a Ganzfeld dome and remained in complete darkness for 5 min before initiating the experiment.

Half millisecond square flashes for scotopic measurements were produced by cyan light emitting diodes of 503 nm peak wavelength. The output of the LED flashes were calibrated using a radiometer (ILT1700 International Light, MA) with a photodiode sensor and scotopic filter that provided readout in the unit of scot cd^*^s/m^2^. These were converted to the unit of photoisomerizations/rod (R^*^/rod) where 1 scot cd^*^s/m^2^ = 581 photoisomerizations/rod/s as previously reported (Saszik et al., 2002; Abd-El-Barr et al., 2009; Tse et al., 2015). The sensor was placed inside the Ganzfield sphere at the level of the mice. By measuring the radiometer readout at various levels of LED input, a standard curve showing the relationship between the LED input and output was obtained.

At the lowest intensity, 25 responses were averaged with a delay of 2 seconds between each flash. As the intensity of the flash increased, fewer responses were averaged with a longer delay between flashes as described in Abd-El-Barr et al. (2009) Tse et al. (2015). At the end of the scotopic recordings, a pair of 1500W xenon lamps (Novatron, Dallas, TX) attenuated with apertures and diffusers were used to produce two saturating light stimuli. Rod ERGs were acquired by single flash stimuli with a strength below the operative range of cones, whereas cone ERGs were measured by a paired-flash protocol using xenon flashes (Pennesi et al., 2003; Abd-El-Barr et al., 2009; Tse et al., 2015). In the paired-flash protocol, an initial conditioning flash (4.6 × 10^6^ R^*^ per M cone) saturates both rods and cones 2 seconds before a probe flash. The ERG recorded by the probe flash (1.8 × 10^6^ R^*^ per M cone) is attributed to responses driven by cones because cones recover faster than rods (Tse et al., 2015).

Signals were amplified with a Grass P122 amplifier (Grass Instruments, West Warwick, RI) and band-pass filtered from 0.1 to 1,000 Hz. Data was acquired with a National Instruments data acquisition unit (USB-6216, National Instruments, TX) at a 10 kHz sampling rate. Traces were averaged and analyzed using a custom Matlab code (MathWorks, Natick, MA) used previously in Abd-El-Barr et al. (2009) Tse et al. (2015). To remove oscillatory potentials before fitting, the scotopic b-wave was digitally filtered using the *filtfilt* function in Matlab (low-pass filter; Fc = 60 Hz). The a-wave was measured from baseline to trough of the initial negative deflection and the b-wave was measured from the a-wave trough to the peak of the subsequent positive deflection (Tse et al., 2015). The relationship between b-wave amplitude and stimulus intensity was described using the saturating hyperbolic Naka-Rushton equation and the Solver function in MS Excel where Bmax is the saturated scotopic b-wave amplitude and I_0.5_ is the stimulus intensity that provides half saturation (Naka and Rushton, 1966; Abd-El-Barr et al., 2009; Tse et al., 2015)_._ Statistical significance was determined using the Holm-Sidak method for multiple comparisons or an unpaired Student's *t*-test using the GraphPad software. Alpha level is set at *p* < 0.05.

## Results

### Nfasc Is Expressed in the Emerging OPL During Synapse Formation

To identify novel cell adhesion molecules that guide synapse formation in the outer retina, we analyzed published RNA sequencing data for unique sets of molecules expressed during synaptogenesis (Sarin et al., 2018). We found the L1-family member, Neurofascin (Nfasc) to be expressed from P7 to P30 in outer retinal neurons (Sarin et al., 2018). To confirm this expression data, we performed antibody staining on retinal sections at various developmental time points: P5, P7, P13, and P30. We found Nfasc begins to be highly expressed in the nascent OPL starting at P7 and continues to adult stages (Figure 1B). By P30, synapse formation in the outer retina is largely complete. At this stage, rod and cone photoreceptors occupy distinct layers within the OPL where they synapse to their respective targets within different sublaminae (Sarin et al., 2018). To determine the sublamina where Nfasc is localized, we co-stained retinal sections with different antibodies to label rod terminals (anti-PSD-95), cone terminals (anti-CAR), cone bipolars (anti-Scgn), rod bipolars (anti-PKC), and horizontal cells (anti-Calb). Interestingly, we found Nfasc mainly overlaps with processes from cone bipolars, rod bipolars, and horizontal cells, and very little with rod and cone terminals as depicted with yellow dotted lines (Figure 1C). These data show Nfasc emerges during synapse formation in the nascent OPL and localizes to processes of postsynaptic neurons (i.e., rod bipolars, cone bipolars, horizontal cells) that synapse selectively to rod and cone photoreceptors.

### Partial Loss of Nfasc in Nfasc CKO Transgenic Mice

**Figure 2:**
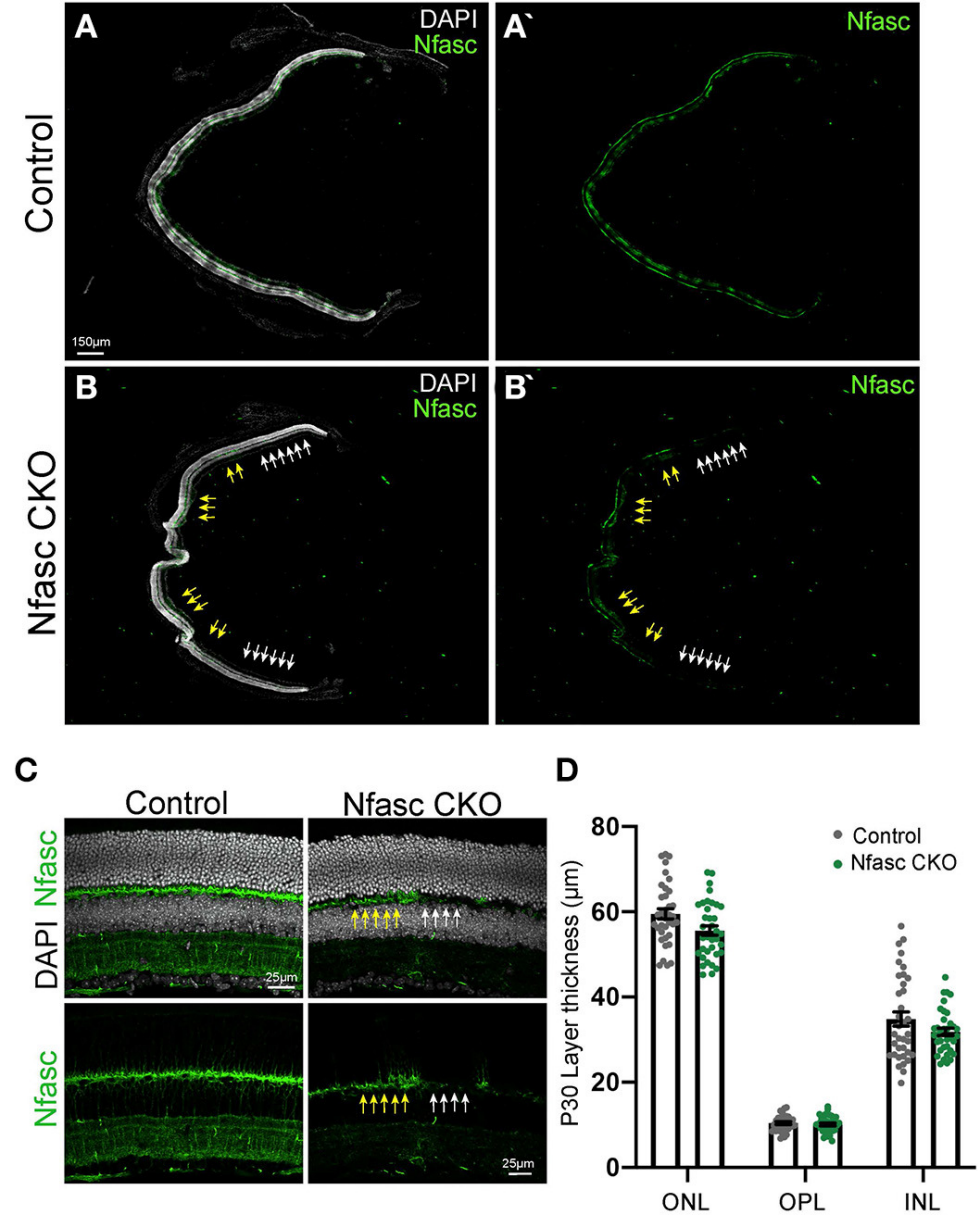
Incomplete loss of Nfasc protein expression in Nfasc CKO **(A–D)**. Representative retinal sections of controls **(A,A')** and Nfasc CKO **(B,B')** taken with a 10X objective and the tiling confocal scanning feature. Nfasc protein expression (anti-Nfasc, green) is absent in a mosaic columnar manner (white arrows) throughout the retina in Nfasc CKO where there are remaining areas with Nfasc expression (yellow arrows). Nuclei is stained with DAPI. Higher magnification of retinal sections is shown in **(C)**. Measurements of retinal layer thickness represented as mean values in controls (*n* = 36 retinal sections from four different animals) in black and Nfasc CKO (*n* = 36 retinal sections from four animals) in green at P30 **(D)**. Scale bar shown in **(A,C)**.

To determine the role of Nfasc in retinal connectivity, we conditionally removed Nfasc in early retinal progenitors by crossing *Chx10-cre* transgenic mice (Rowan and Cepko, 2004) to a Nfasc floxed allele (*Nfasc*^*flox*/*flox*^) (Amor et al., 2017). This line is referred to as Nfasc CKO. We performed antibody staining to validate loss of Nfasc in transgenic adult mice (Figures 2A–C). Low magnification of a representative retinal section shows Nfasc is removed in a mosaic manner in Nfasc CKO where there are areas devoid of Nfasc (white arrows) immediately adjacent to areas with remaining Nfasc expression (yellow arrows) (Figure 2B). This expression pattern is distinct from controls that display uniform Nfasc expression throughout the OPL (Figure 2A). We also noticed that Nfasc was consistently removed in the central-peripheral region of the retina (white arrows). Prior studies using *Chx10-cre* mice have reported a similar mosaic expression pattern in the retina where there are a few cells that fail to recombine (Lefebvre et al., 2008). Consistent with these findings, we also observe a similar mosaic or partial knock out of Nfasc in Nfasc CKO animals. As Nfasc was consistently reduced in the central-periphery region of the retina, we performed all our subsequent analysis in these areas.

We then performed *in situ* hybridization in wild-type retinas to address which cell-type expresses Nfasc during synapse formation (i.e., P13). *Nfasc* mRNA appears to be highly expressed in the top-most region of the INL (Figure 1D) where the cell bodies of horizontal cells, rod bipolars, and cone bipolars reside. Using Nfasc CKO retinas, we then addressed in which cell-type were we knocking down Nfasc by performing *in situ* hybridization followed by antibody staining. Similar to wild-type retinas, *Nfasc* mRNA localizes to the cell bodies of rod bipolars (anti-PKC), cone bipolars (anti-Scgn), and horizontal cells (anti-Calb) in controls (Figure 1D). Interestingly, *Nfasc* mRNA appears to be significantly reduced in rod bipolars compared to controls. However, *Nfasc* mRNA levels in cone bipolars and horizontal cells do not appear to be affected (Figure 1D). To confirm these findings, we counted the number of *Nfasc* mRNA puncta (~0.6 μm in size) within rod bipolars, cone bipolars, and horizontal cells. We found that indeed there is a significant reduction in the number of Nfasc puncta in rod bipolars (controls: 3.5 ± 2.2 puncta from 28 rod bipolars; Nfasc CKO: 1.93 ± 0.78 puncta from 30 rod bipolars) but not in cone bipolars (controls: 2.07 ± 1 puncta from 14 cone bipolars; Nfasc CKO: 1.95 ± 1.1 puncta from 39 cone bipolars) nor horizontal cells (controls: 3.3 ± 2.27 puncta from 20 horizontal cells; Nfasc CKO: 3.61 ± 2.15 puncta from 18 horizontal cells). The remaining *Nfasc* expression we observe in Nfasc CKO retinas could be due to partial knockdown of Nfasc as seen with antibody staining, or non-specific binding of our *in situ* hybridization probe.

### Disruption of Nfasc Results in Selective Reduction of Synaptic Proteins in Rods

**Figure 3:**
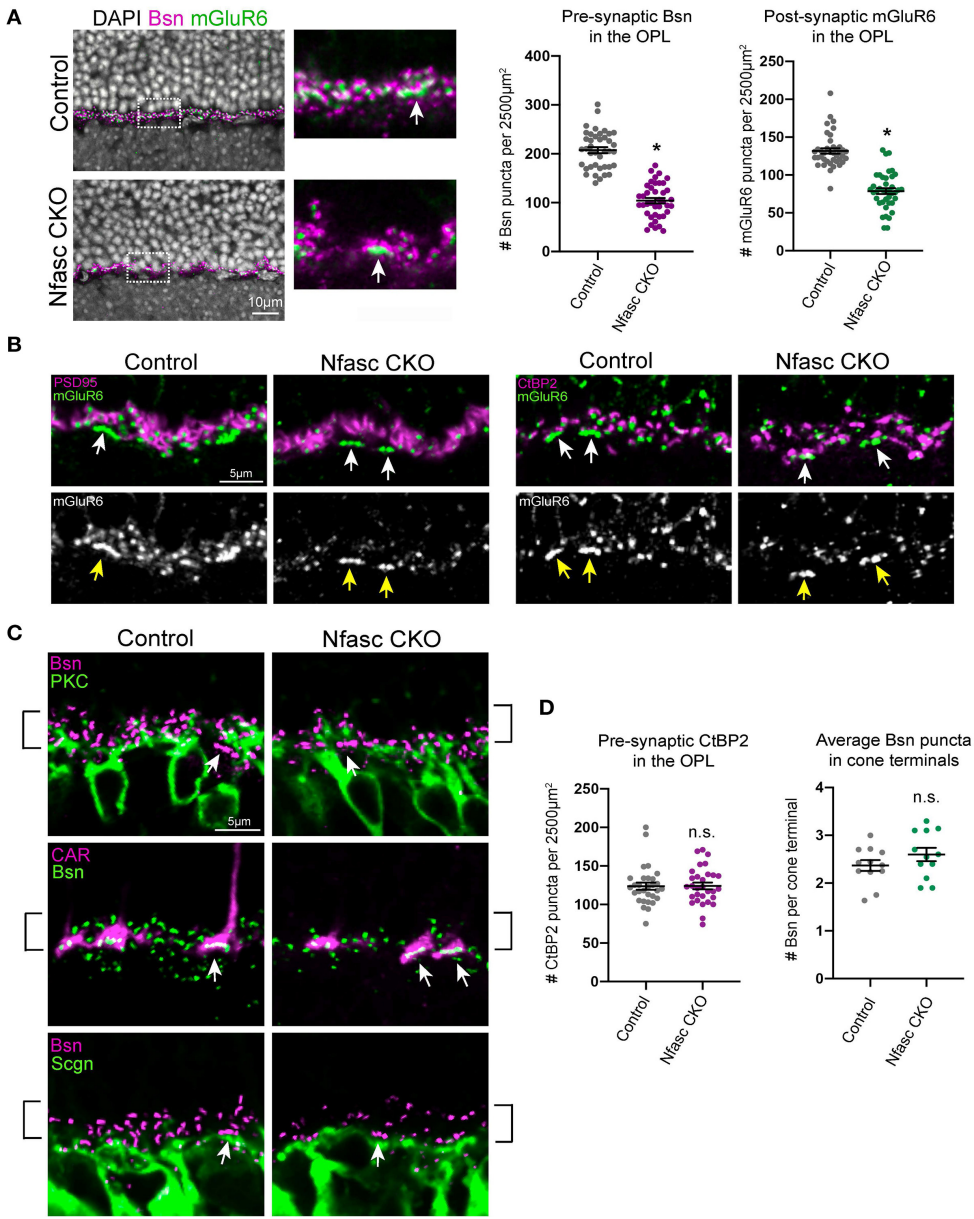
Selective reduction of pre- and post-synaptic protein expression in Nfasc CKO **(A–D)**. Pre-synaptic Bassoon (magenta) and post-synaptic mGluR6 (green) protein is significantly reduced in Nfasc CKO compared to controls **(A)**. Insets are zoomed images of dotted boxed region. At P30, Bsn and mGluR6 forms a puncta-like structure with 0.6 and 1.0 μm in size, respectively. Number of Bsn and mGluR6 puncta were counted in the OPL with an area of ~2,500 μm^2^. A total of 10 different confocal sections were analyzed from four control animals (*n* = 40) and four Nfasc CKO animals (*n* = 40). Data are represented as mean values ± SEM. Statistical significance was determined using an unpaired *t*-test. *p < 0.0001. Images are shown as a single confocal section. Scale bar, 10 μm **(A)**. Rod terminals appear to be normally positioned in the OPL in Nfasc CKO **(B)**. Staining with anti-PSD-95 (magenta) and anti-CtBP2 (magenta) shows expression is not affected even in areas where mGluR6 expression is reduced (green, white). Cone terminals appear to be unaffected as denoted by white and yellow arrows **(B)**. Dendrites of rod bipolars (anti-PKC, green) and cone bipolars (anti-Scgn, green) are localized to the OPL in both Nfasc CKO and control retinas. Bsn puncta (magenta, green) appears to be reduced in the rod synaptic layer as outlined by black brackets and not in the cone synaptic layer as seen with anti-CAR (magenta) and depicted with white arrows **(C)**. Quantification of CtBP2 puncta within the OPL shows no statistical difference between Nfasc CKO and controls. The average number of Bsn puncta per cone terminal within a confocal section are plotted. These measurements were taken from 12 different retinal sections from four control and four Nfasc CKO mice. A total of 118 cone terminals were analyzed for controls and 101 for Nfasc CKO **(D)**.

As rod photoreceptors make synapses with rod bipolars, we next investigated if loss of Nfasc results in synaptic defects between these two synaptic partners. To perform this analysis, we used available antibodies that label pre-synaptic proteins such as Bassoon (Bsn) and post-synaptic proteins such as the metabotropic Glutamate Receptor 6 (mGluR6). In wild-type retinas, Bsn is found at the terminals in both rod and cone photoreceptors, and mGluR6 accumulates at the dendrites of both rod bipolars and ON cone bipolars (Cao et al., 2015). At P30, both Bsn and mGluR6 form a puncta-like structure within the OPL that are in juxtaposition to one another. The number of Bsn and mGluR6 were quantified from retinal sections that were imaged using a 40X objective with an OPL area of ~2,500 μm^2^. Our data revealed a significant reduction in pre-synaptic Bsn puncta along the OPL (49.8%) and a 40.2% reduction of post-synaptic mGluR6 puncta (Figure 3A). This decrease in expression was not simply due to cell loss as measurements of each layer thickness within the outer retina (ONL, OPL, INL) showed no statistical difference between Nfasc CKO and controls (Figure 2D). A closer examination of the OPL in Nfasc CKO animals revealed that Bsn and mGluR6 were selectively reduced in the upper sublamina where rods synapse with rod bipolars and not in the lower sublamina where cones synapse with cone bipolars (white arrows) (Figure 3A). In wild-type retinas, Bsn appears as puncta-like structures localized to the dendritic tips of rod bipolars (anti-PKC) whereas in cone bipolars (anti-Scgn) about 3-4 Bsn puncta cluster at the base of the dendrites (Figure 3C). Bsn clustering in cone terminals can also be seen with co-staining with the cone marker (anti-CAR) as shown in Figure 3C. Consistent with Figure 3A, Bsn appears to be significantly reduced in rod synaptic layer (i.e., upper sublamina of the OPL) compared to the cone synaptic layer (i.e., lower sublamina of the OPL). This observation was confirmed by counting the number of Bsn puncta (~0.6 μm in size) in cone terminals labeled with CAR, which showed no statistical difference between Nfasc CKO (2.58 ± 1.30 Bsn puncta per cone) and controls (2.31 ± 0.96 Bsn puncta per cone) using an unpaired Student's *t*-test with a *p* < 0.05 (Figure 3D). A total of 118 cone terminals were analyzed for controls (*n* = 4 mice) and 101 for Nfasc CKO (*n* = 4 mice). Moreover, reduction of Bsn puncta do not appear to be due to rod bipolar dendrites failing to reach the OPL as PKC staining shown in Figure 3C reveals them to be normally positioned within the OPL.

Remarkably, other pre-synaptic proteins that are localized to photoreceptor terminals do not appear to be reduced in animals with loss of Nfasc. Antibodies against PSD-95 (a pre-synaptic marker highly expressed in rod terminals) and CtBP2 (a pre-synaptic marker for both rods and cones) show no significant decrease in protein expression even in areas where mGluR6 is significantly reduced (Figure 3B). This finding was confirmed by counting the number of CtBP2 puncta (0.7 μm in size) in retinal sections within an OPL area of ~2,500 μm^2^ in Nfasc CKO (123.7 ± 25.43 CtBP2 puncta) and controls (124.1 ± 23.73 CtBP2 puncta) and shown to not be statistically significant using an un-paired *t*-test, *p* < 0.05 (Figure 3D). These data suggests that rod terminals may be positioned correctly within the OPL in Nfasc CKO retinas as noted with PSD95 and CtBP2 expression. However, they fail to form or maintain synapses with rod bipolars as seen with loss of Bsn and mGluR6.

### Transmission Electron Microscopy (TEM) to Visualize Rod Terminals

**Figure 4:**
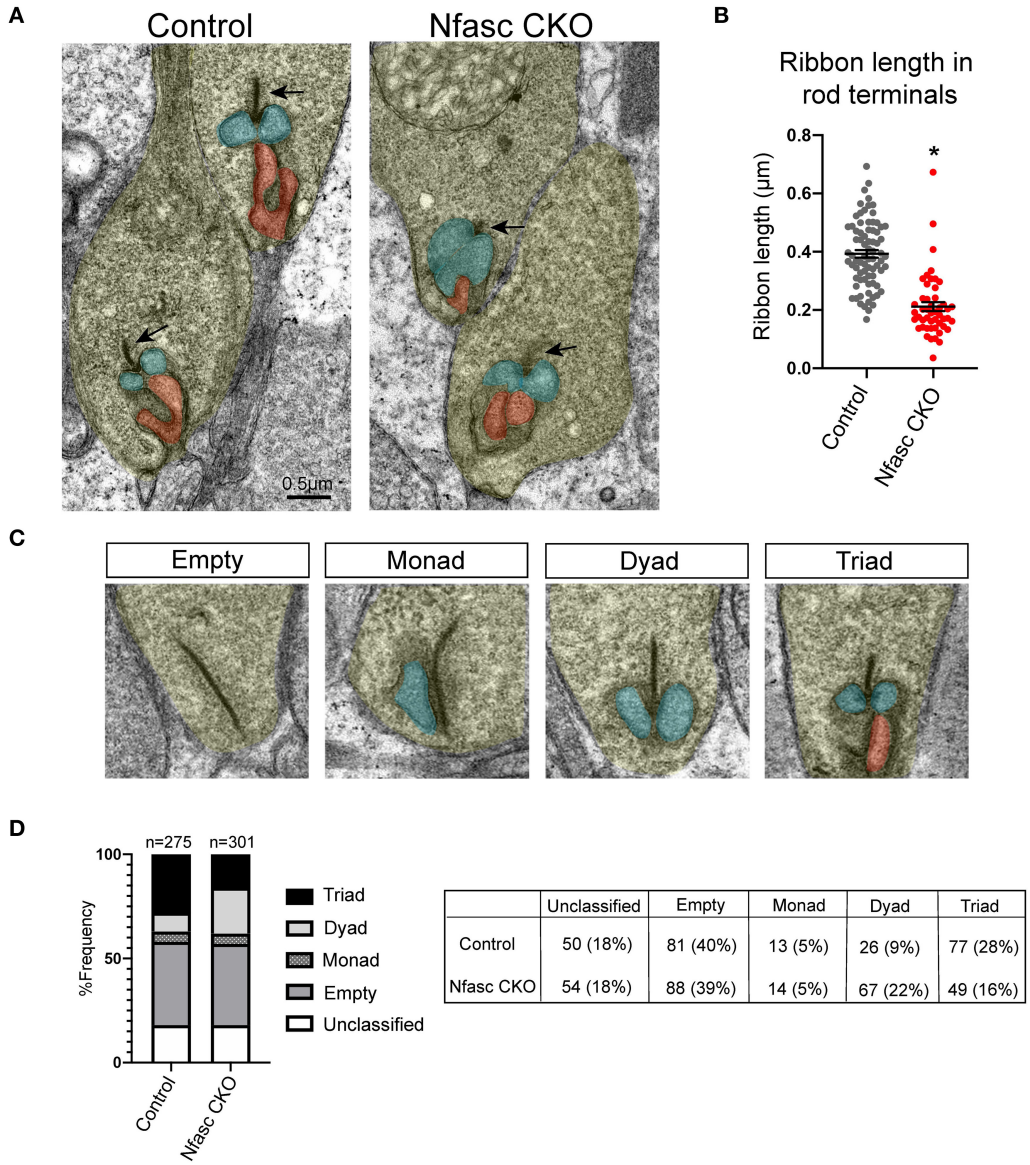
Transmission Electron Microscopic (TEM) analysis reveals synaptic defects in Nfasc CKO **(A–D)**. Representative electron micrographs of rod terminals of controls and Nfasc CKO mice at P30 **(A)**. In controls, rod terminals (yellow) contain large, well-defined ribbon synapses (arrows) with invaginations from horizontal cells (blue) and ON bipolar cells (red). In contrast, Nfasc CKO display short and poorly defined ribbon synapses (arrows). Quantification of the length of ribbon synapses in controls (77 rod terminals; *n* = 4 mice) and Nfasc CKO (49 rod terminals; n=4 mice). Data represented as mean ± SEM. Statistical significance determined by an unpaired *t*-test. *p < 0.0001 **(B)**. Rod terminals were designated as unclassified, empty, monad, dyad, or triad **(C)**. A total of 275 rod terminals were analyzed for controls (*n* = 4 mice) and 301 rod terminals for Nfasc CKO (*n* = 4 mice). Frequency (%) shown for controls and Nfasc CKO in **(D)**. Exact values are listed in the table. Scale bar = 0.5 μm.

To further examine the rod synaptic structure, we performed TEM on retinas from Nfasc CKO and control mice at P30 (Figure 4). We classified photoreceptor terminals into four categories as previously described (Nemitz et al., 2019): [1] empty terminals - no visible processes, [2] monad – only one invaginating horizontal cell process, [3] dyad - two invaginating horizontal cell processes, and [4] triad - two invaginating horizontal cell processes with at least one ON bipolar dendrite (Figure 4C). Our data revealed a shift in the frequency of dyads compared to triads in Nfasc CKO compared to controls but not in empty, monads, or unclassified. We analyzed 275 rod terminals from *n* = 4 mice for controls and found a frequency distribution of 18% unclassified, 40% empty, 5% monad, 9% dyad, and 28% triad. For Nfasc CKO, we analyzed 301 rod terminals from *n* = 4 mice and found the frequency to be 18% unclassified, 39% empty, 5% monad, 22% dyad, and 16% triad. See Figure 4D. The number of dyads and triads in Nfasc CKO compared to controls was determined to be significant using an unpaired Student's *t*-test (dyads: *p* = 0.0003; triads: *p* = 0.04) but not for the other categories (*p* < 0.05 considered as significant). The shift in the frequency of triads to dyads seen with loss of Nfasc is consistent with dendrites of rod bipolars failing to make or maintain synapses with rod photoreceptors. We next addressed if the remaining triad structures were formed properly by examining the ribbon synapse. By P30, photoreceptors form a unique electron-dense structure called the ribbon synapse (Regus-Leidig et al., 2009). The ribbon synapse is the active zone of neurotransmitter release comprised of hundreds of vesicles and scaffolding proteins such as Bassoon (Dick et al., 2003; Regus-Leidig et al., 2009). We observed that ribbon synapses (black arrows) in Nfasc CKO were abnormal in shape and size compared to controls (Figure 4A). To confirm this observation, we measured the ribbon length from the remaining triad structures in controls (77 rod terminals; *n* = 4 mice) and Nfasc CKO (49 rod terminals; *n* = 4 mice). Ribbon length was significantly reduced in Nfasc CKO (0.21 μm ± 0.11) compared to controls (0.39 μm ± 0.11) using an un-paired Student's *t*-test with a *p* < 0.0001 (Figure 4B). These findings show that there are less rod bipolars forming proper synapses with rod terminals which further supports a role for Nfasc at the rod synapse.

### Loss of Nfasc Leads to Reduced Rod-Driven Retinal Responses

**Figure 5:**
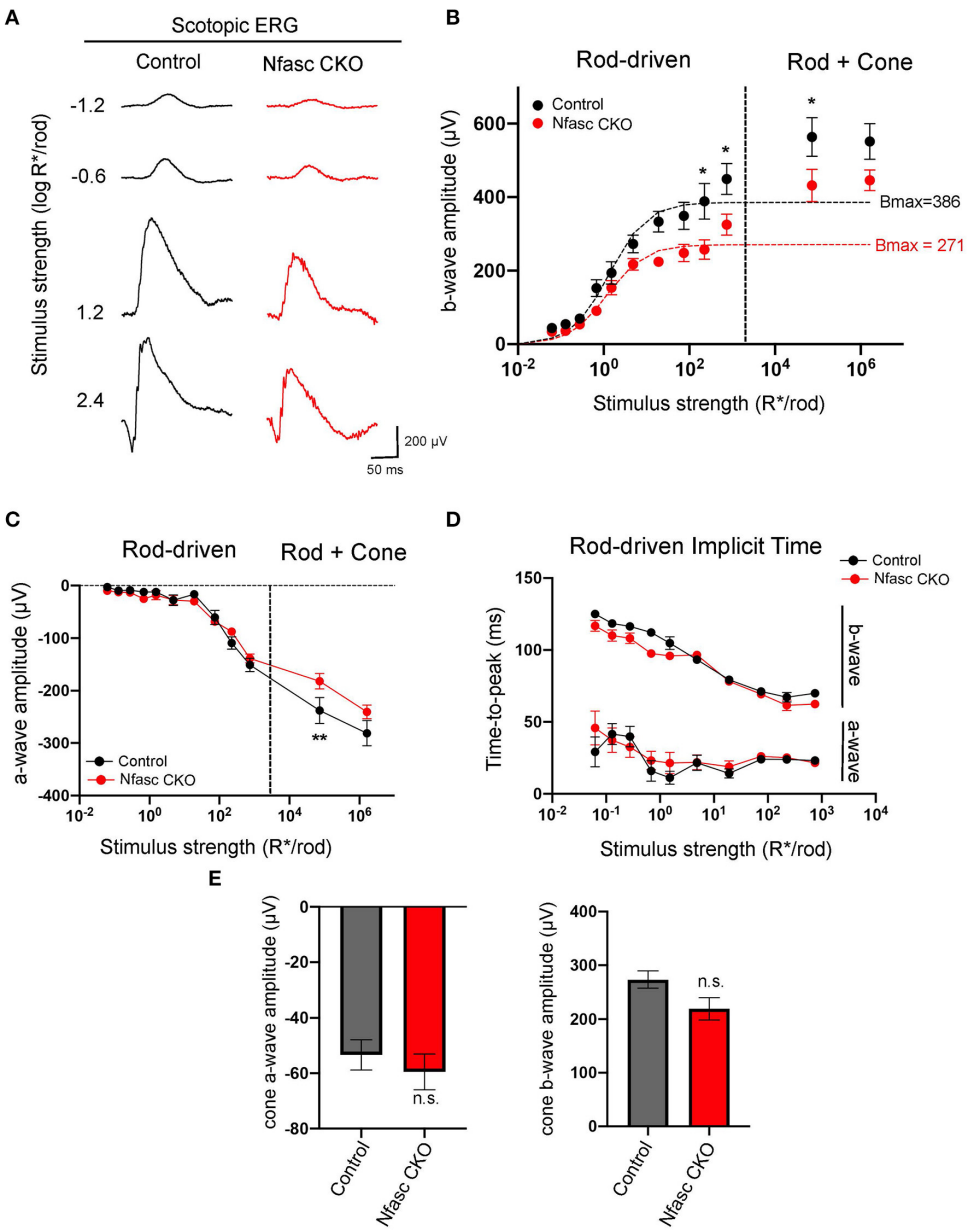
Electroretinograms (ERG) from Nfasc CKO mice **(A–E)**. ERG traces of control (black) and Nfasc CKO (red) mice at different scotopic (rod-driven) stimulus intensities **(A)**. Stimulus response plot showing b-wave amplitudes of dark-adapted control (black, *n* = 8 from four mice) and Nfasc CKO (red, *n* = 8 from four mice). Data points within the rod operative range are fitted with a hyperbolic saturating curve using the Naka-Rushton equation. Dotted line denotes the approximate threshold for cone photoreceptors **(B)**. Stimulus response plot of scotopic a-wave amplitudes of control (black, *n* = 8 from four mice) and Nfasc CKO (red, *n* = 8 from four mice) **(C)**. Implicit times for scotopic ERG a-wave and b-wave responses as a function of stimulus intensity **(D)**. Isolated amplitudes of the cone a-wave and b-wave using the paired flash method **(E)**. Data represented as mean values ± SEM. Statistical significance determined by Holm-Sidak method for multiple comparisons. *p < 0.05, **p < 0.001.

To further validate that loss of Nfasc disrupts rod-to-rod bipolar connectivity, we measured retinal responses by performing *in vivo* full-field electroretinograms (ERG) responses in dark-adapted mice. Mice were exposed to flashes of varying light intensities separated into four light zones as described in Abd-El-Barr et al. (2009): zone I (< 0.1 photoisomerizations/rod), zone II (between 0.1 and 30 photoisomerizations/rod), zone III (between 30 and 10^3^ photoisomerizations/rod), and zone IV (> 10^3^ photoisomerizations/rod). Photopic responses were elicited using a paired-flash protocol. Individual ERG traces of Nfasc CKO (red) compared to controls (black) show a slight decrease in b-wave responses compared to controls under scotopic (rod-driven) conditions (Figure 5A). To confirm this observation, we plotted b-wave responses fitted with the Naka-Rushton equation and calculated the maximum rod-driven b-wave response (i.e., Bmax) as well as the stimulus intensity that provides half saturation (I_0.5_). Our data revealed Nfasc CKO mice had a Bmax value of 271 μV with an I_0.5_ of 0.09 log R^*^/rod (red dotted line) which was 30% lower than controls that had a Bmax of 386 μV with an I_0.5_ of 0.14 log R^*^/rod (black dotted line) (Figure 5B). We found that rod-driven responses were statistically significant at higher stimulus intensities and not at lower as determined by the Holm-Sidak method for multiple comparisons (*p* < 0.05). This could be due to the partial knockout we observe in Nfasc CKO where only at higher light intensities when more rods are recruited there is a significant difference in b-wave responses. In addition, we also noticed that the first flash to elicit cone responses is significant but not the second flash. This could be attributed to some remaining rod responses that failed to become saturated with the first flash. To determine this, we isolated cone b-wave responses and found no statistical difference using an unpaired Student's *t*-test (*p* < 0.05) between Nfasc CKO and controls (Figure 5E). Next, we examined a-wave responses from Nfasc CKO and control mice. We found a-wave responses under rod-driven conditions are not statistically significant using the Holm-Sidak method (*p* < 0.05) but also found that the first flash under the paired protocol to be significant (Figure 5C). Similar to the cone b-wave responses, we isolated cone a-wave responses and found no statistical difference between Nfasc CKO and controls (Figure 5E). Interestingly, although we find a reduction in scotopic b-wave responses, the time-to-peak or implicit time are not affected in a-wave nor b-wave responses (Figure 5D). These data suggest that rod-driven bipolar responses (i.e., scotopic b-wave) and not cone-driven responses (photopic b-wave) are affected due to loss of Nfasc. The slight reduction in ERG b-wave response (~30%) could be attributed to the partial knockdown of Nfasc in Nfasc CKO animals as mentioned previously, or redundant pathways that can restore normal physiological responses in the absence of Nfasc.

## Discussion

In the present study, we identified a novel role for the Nfasc cell adhesion molecule in rod photoreceptor synapses in the mouse outer retina. We found Nfasc to be localized to the synaptic layer and highly expressed in rod bipolars. Moreover, disruption of Nfasc results in failure to form or maintain proper synapses in the rod pathway. This was seen with selective loss of pre-and post-synaptic protein expression in the rod synaptic layer, a decrease in ribbon synapses at rod terminals, and abnormal rod-driven retinal responses. In summary, our work begins to elucidate a new molecular pathway involved in rod photoreceptor connectivity.

### Nfasc Function at the OPL

Over the last years, several cell adhesion molecules have been implicated at the rod photoreceptor synapse (Hoon et al., 2014; Zhang et al., 2017; Sanes and Zipursky, 2020). Some of these appear to serve a structural function where loss of these molecules disrupts the physical contacts between photoreceptors and their synaptic partners such as in the case of Ngl2^−/−^ and SynCAM1^−/−^ mutant mice (Soto et al., 2013; Ribic et al., 2014). Others appear to serve more of a functional role where physical contacts between synaptic partners are not as affected and yet transmission is greatly impaired like in loss of Pikachurin and Dystroglycan (Omori et al., 2012; Orlandi et al., 2018). And others such as Elfn1 appear to be involved in both the physical and functional aspect of photoreceptor synapses where they recruit key components to the synapse for proper transmission (Cao et al., 2015; Wang et al., 2017). Our data on Nfasc suggests that this cell adhesion molecule may be involved in the synaptic integrity of rod photoreceptors. We found that rod terminals and the dendrites appear to be normally positioned within the OPL; however, key synaptic components such as Bassoon and mGluR6 fail to be expressed in the rod synaptic layer. Electron microscopic analysis of rod terminals also supports that synapses are abnormal in Nfasc CKO retinas, as ribbon synapses (comprised of Bassoon protein) appear smaller in length compared to controls. Although synapses in Nfasc CKO appear compromised, they still are largely able to function based on ERG recordings except at higher scotopic light intensities. This could be due to several reasons. First, we noticed that Nfasc CKO animals are not a complete knockout for Nfasc, as the OPL still retinas some Nfasc expression. This remaining expression could be sufficed to elicit normal rod-driven responses at lower stimulus intensities but not at higher intensities where more rods are recruited. This could be addressed using germline knock outs for Nfasc however previous studies show that these animals die shortly after birth (Sherman et al., 2005). Second, other cell adhesion molecules may work in parallel pathways to ensure proper formation of rod synapses. The cell adhesion molecule Elfn1 found in rods has been shown to bind trans-synaptically to mGluR6 in rod bipolars and directly recruit it to the OPL (Cao et al., 2015). Disruption of Elfn1 results in complete loss of mGluR6 at the rod synaptic layer but not in the cone synaptic layer. We found Nfasc is not in rods but highly expressed in rod bipolars. This raises the question if Elfn1 and Nfasc act in a similar manner where Elfn1 in rods recruit key components from rod bipolars, whereas Nfasc in rod bipolars recruits pre-synaptic proteins such as Bassoon either directly or indirectly from rods. Third, although Nfasc begins to be expressed at early stages during OPL development (i.e., P7), Nfasc may have more of a later role at maintaining synapses. Based on the timing of Nfasc expression, we focused our analysis at P30 shortly after synapse formation is complete. The Nfasc CKO phenotype may begin to emerge at P30 but may not be fully done until several months later. This is often seen with several models of retinal degeneration where phenotypes are not seen until many months after synapse formation (Chang et al., 2002). Thus, examining the retinas from Nfasc CKO at older stages will help answer this question. Future work will be needed to further elucidate the role of Nfasc at the rod synapse along with the interactions of other cell adhesion molecules.

### Nfasc Binding Partners in Other Retinal Neurons

Nfasc is known to bind in both a homophilic and heterophilic manner to mediate cell-to-cell interactions (Eshed et al., 2005, 2007; Wei and Ryu, 2012). In other regions of the nervous system, Nfasc is known to bind to other cell adhesion molecules to facilitate molecular interactions between glia and neurons (Eshed et al., 2005, 2007; Sherman et al., 2005; Rasband and Peles, 2015). These Nfasc-mediated interactions are required for the precise positioning and formation of Nodes of Ranvier within myelinated axons (Davis et al., 1996; Sherman et al., 2005; Rasband and Peles, 2015). Our data on Nfasc could have a similar role in mediating key neuron-neuron interactions. Thereby, we speculate that Nfasc along with other cell adhesion molecules may be required for proper retinal circuit formation. Recent RNA sequencing data shows Nfasc is differentially expressed among photoreceptors throughout synapse formation (P7-30), where Nfasc transcript levels are higher in rods compared to cones (Sarin et al., 2018). However, our *in situ* hybridization data in wild-type retinas at P13 shows low levels of *Nfasc* mRNA transcripts in the ONL where rods reside. Removal of Nfasc in a cell-type specific manner may be necessary to determine if Nfasc in rods binds in a homophilic manner to rod bipolars. Moreover, RNA sequencing data reveals known binding partners of Nfasc such as Neuronal cell adhesion molecule (Nrcam) and Contactin 1 (Cntn1) are expressed in outer retinal neurons (Rasband and Peles, 2015; Sarin et al., 2018). Thus, disruption of these cell adhesion molecules may elucidate complex molecular interactions of how Nfasc mediates photoreceptor connectivity.

### Site of Action of Nfasc Function

Our data demonstrates that loss of Nfasc results in phenotypes that are consistent with disruption of rod-to-rod bipolar connectivity. However, the axon of horizontal cells also forms synaptic connections to rod terminals prior to rod bipolars (Kolb, 1974). Recent data shows horizontal cells are required for the dendrites of rod bipolars to form synapses with rod photoreceptors (Nemitz et al., 2019). Ablation of horizontal cells at early stages results in dendrites of rod bipolars failing to express post-synaptic markers such as mGluR6 and failing to innervate rod terminals (Nemitz et al., 2019). These findings are consistent with the phenotypes we observe in Nfasc CKO where there is a decrease in mGluR6 expression and rod synapses either fail to form or maintain synapses with rod bipolars. Thus, Nfasc from rod bipolars could be the key molecule that interacts with horizontal cells to facilitate rod synapse formation. However, dendrites of rod bipolars also misproject into the ONL with removal of horizontal cells at both early and later stages of development (Keeley et al., 2013; Nemitz et al., 2019). This phenotype is not seen with loss of Nfasc nor is this seen with loss of Elfn1 (Cao et al., 2015) which suggests that other horizontal cell-mediated mechanisms may be responsible for proper positioning of rod bipolar dendrites to the OPL. The differences in phenotypes suggests that horizontal cells express other molecules that perform multiple functions during synapse formation.

Taken together, we uncovered a new role for the cell adhesion molecule Nfasc in rod photoreceptors within the outer retina. Future studies will reveal the cell-type specific requirements of Nfasc function along with the binding partners that facilitate key neuron-neuron interactions.

## Data Availability Statement

The raw data supporting the conclusions of this article will be made available by the authors, without undue reservation.

## Ethics Statement

The animal study was reviewed and approved by Baylor College of Medicine Institutional Animal Care and Use Committee.

## Author Contributions

SP, DG-S, and EZ-S designed the experiments and wrote the manuscript. DG-S and SP performed histological staining. DG-S carried out the RNAscope experiments. SP and EZ-S acquired the confocal images and performed quantification. EZ-S performed the ERG experiments. All authors contributed to this work and approved the final version of the manuscript.

## Conflict of Interest

The authors declare that the research was conducted in the absence of any commercial or financial relationships that could be construed as a potential conflict of interest.
